# Impact of the Timing of Mechanical Circulatory Support on the Outcomes in Myocardial Infarction-Related Cardiogenic Shock: Subanalysis of the PREPARE CS Registry

**DOI:** 10.3390/jcm13061552

**Published:** 2024-03-08

**Authors:** Dan M. Prunea, Eva Bachl, Lukas Herold, Sadeek S. Kanoun Schnur, Sascha Pätzold, Siegfried Altmanninger-Sock, Gudrun A. Sommer, Theresa Glantschnig, Ewald Kolesnik, Markus Wallner, Klemens Ablasser, Heiko Bugger, Eva Buschmann, Andreas Praschk, Friedrich M. Fruhwald, Albrecht Schmidt, Dirk von Lewinski, Gabor G. Toth

**Affiliations:** 1 Department of Cardiology, Medical University of Graz, 8010 Graz, Austria; d.prunea@gmail.com (D.M.P.); eva.bachl@stud.medunigraz.at (E.B.); sid.cannon@doctors.org.uk (S.S.K.S.); andreas.praschk@medunigraz.at (A.P.); dirk.von-lewinski@medunigraz.at (D.v.L.); 2 “Niculae Stăncioiu” Heart Institute, University of Medicine and Pharmacy “Iuliu Hațieganu”, 400347 Cluj-Napoca, Romania; 3 Doctoral School of Clinical Medicine, Faculty of Medicine, University of Szeged, 6720 Szeged, Hungary; 4 Royal Devon University Healthcare NHS Foundation Trust, Exeter EX2 5DW, UK

**Keywords:** cardiogenic shock, mechanical circulatory support, myocardial infarction, mechanical circulatory support timing, in-hospital mortality

## Abstract

(1) **Background**: Mechanical circulatory support (MCS) in myocardial infarction-associated cardiogenic shock is subject to debate. This analysis aims to elucidate the impact of MCS’s timing on patient outcomes, based on data from the PREPARE CS registry. (2) **Methods**: The PREPARE CS prospective registry includes patients who experienced cardiogenic shock (SCAI classes C–E) and were subsequently referred for cardiac catheterization. Our present analysis included a subset of this registry, in whom MCS was used and who underwent coronary intervention due to myocardial infarction. Patients were categorized into an Upfront group and a Procedural group, depending on the timing of MCS’s introduction in relation to their PCI. The endpoint was in-hospital mortality. (3) **Results**: In total, 71 patients were included. MCS was begun prior to PCI in 33 (46%) patients (Upfront), whereas 38 (54%) received MCS during or after the initiation of PCI (Procedural). The groups’ baseline characteristics and hemodynamic parameters were comparable. The Upfront group had a higher utilization of the Impella^®^ device compared to extracorporeal membrane oxygenation (67% vs. 33%), while the Procedural group exhibited a balanced use of both (50% vs. 50%). Most patients suffered from multi-vessel disease in both groups (82% vs. 84%, respectively; *p* = 0.99), and most patients required a complex PCI procedure; the latter was more prevalent in the Upfront group (94% vs. 71%, respectively; *p* = 0.02). Their rates of complete revascularization were comparable (52% vs. 34%, respectively; *p* = 0.16). Procedural CPR was significantly more frequent in the Procedural group (45% vs. 79%, *p* < 0.05); however, in-hospital mortality was similar (61% vs. 79%, respectively; *p* = 0.12). (4) **Conclusions**: The upfront implantation of MCS in myocardial infarction-associated CS did not provide an in-hospital survival benefit.

## 1. Introduction

Cardiogenic shock (CS) is characterized by a significant reduction in cardiac output, leading to inadequate end-organ perfusion and resulting in multiorgan failure, and it is consequently associated with extremely high mortality [1,2]. The predominant etiology of CS is acute myocardial infarction (AMI), which accounts for over 80% of cases and often precipitates a critical dysfunction of the left, the right, or both ventricles. Despite an improved acute phase approach and intensive care management, CS remains the predominant cause of death in patients presenting with AMI [3].

While early revascularization is the main and most effective approach to CS after myocardial infarction, mechanical circulatory devices (MCS) are considered as a last resort for maintaining circulation in refractory CS. Recent trials have not shown any benefit, with limited evidence concerning their indication [4]. The intra-aortic balloon pump (IABP) was the first and most-used MCS device that, although it helped in decreasing the afterload of the left ventricle, had a limited capacity to increase cardiac output, while recent evidence from several recent meta-analyses and randomized controlled trials has questioned the effectiveness of the IABP by showing that there was no benefit to this device [5]. Other MCS systems, such as the VA-ECMO (veno-arterial extracorporeal membrane oxygenation), were developed in ongoing efforts to improve patient outcomes. The utilization of the VA-ECMO has increased considerably during the last decade; however, its potential benefits could be overbalanced by the significant risk of device-related complications. Moreover, patients receiving VA-ECMO devices for CS due to acute MI were associated with poor neurological outcomes and prolonged inpatient care [6].

Data collected from randomized clinical trials regarding the safety, efficacy, and optimal timing of MCS device delivery is scarce and the largest trials, IABP-SHOCK II [5] and ECLS shock [6], failed to prove the superiority of MCS use when considering survival rates. The latter showed a neutral 30-day follow-up regarding its primary (all-cause mortality) and secondary endpoints, such as MI or repeat revascularization. Further safety concerns were raised considering stroke and major bleeding complications, as well as high rates of peripheral ischemic vascular complications and mortality, which really question the usefulness of MCS in MI-related CS patients [7,8].

Yet, despite not having any clear evidence to support its beneficial impact on clinical outcomes, MCS is widely used in the treatment of CS and considered the sole potential tool for maintaining systemic blood perfusion in patients with refractory CS [9]. Despite its capacity to ensure end-organ perfusion until the potential recovery of cardiac function, the delivery of a long-term assist device, or until cardiac transplantation, the utilization of MCS has generated conflicting data across various shock center registries. Nonetheless, these data do suggest that adhering to a standardized, multidisciplinary treatment algorithm for MCS implementation could lead to improved survival rates [10,11]. However, the optimal timing for MCS’s initiation remains uncertain [11].

While RCTs are the best tool for generating scientific evidence, registries have the potential to shed new light on real-world applications. Considering these aspects, the aim of our present analysis was to understand, in the context of real-world settings, whether the timing of an MCS implantation correlates with short-term patient outcomes. Our analysis was performed based on the PREPARE CS registry [12].

## 2. Materials and Methods

The PREPARE CS is a single-center prospective registry that was collected at the University Heart Center Graz, Austria, over a consecutive 4-year period, from May 2019 to April 2023. The registry enrolled all patients with stage C–E cardiogenic shock, as classified by Society for Cardiovascular Angiography and Interventions (SCAI), who were consecutively referred to the cardiac catheterization laboratory. Throughout the observation period, a total of 557 patients were enrolled in the registry. Cardiogenic shock was identified based on criteria indicative of prolonged hypoperfusion and a need for vasoactive medication for maintaining sufficient perfusion pressure [2].

In this analysis we focused on a subset of patients in whom CS was confirmed to be caused by myocardial infarction, indicating PCI, and who received adjunct MCS. According to our center’s practice, the MCS devices incorporated into this study were either the Impella^®^ CP Heart Pump (Impella; Abiomed Inc., Danvers, MA, USA) or a veno-arterial extracorporeal membrane oxygenator (VA-ECMO; Xenios AG, Heilbronn, Germany). Both these devices were delivered via femoral access. Patients receiving an intra-aortic ballon pump (IABP) device were not included within this registry. The appropriate MCS device was chosen according to the ventilation capacity and residual circulation of the patients. In cases of moderate-to-severely impaired circulatory reserves, the Impella^®^ CP Heart Pump was used, while in patients with marked oxygenation insufficiency (indicated by the Horowitz index) or with severely impaired or no circulatory reserve, an VA-ECMO device was implanted.

Patients were categorized into two groups according to the timing of MCS relative to their PCI: the ‘Upfront’ group, receiving MCS prior to revascularization, and the ‘Procedural’ group, receiving MCS at any time after their PCI had started. These analyses’ primary endpoint was in-hospital mortality.

The Ethics Committee of the Medical University of Graz, Austria (EK 31–323 ex 18/19), granted approval for this study, which adheres to the 1964 Declaration of Helsinki and its subsequent revisions. Additionally, this research was conducted in accordance with the guidelines of the International Conference on Harmonization for Good Clinical Practice (ICH GCP E6 guidelines).

### Statistical Analysis

All analyses were performed using Prism GraphPad 9.0 (GraphPad Software Inc., San Diego, CA, USA). Summary descriptive statistics are reported as mean ± standard deviation or *n* (%), as appropriate. Normal distribution was tested by a D’Agostino-Pearson omnibus normality test. Continuous variables were compared by Mann–Whitney tests or Kruskal–Wallis tests, and categorical variables were compared with Fisher’s exact or chi-square tests, as appropriate. Results are expressed in an odds ratio (OR) with a 95% confidence interval (CI). A probability value of *p* < 0.05 was considered significant.

## 3. Results

Between May 2019 and April 2023, 406 patients underwent percutaneous revascularization due to acute myocardial infarction-associated CS (SCAI classes C–E). Among these patients, the MCS devices mentioned above were utilized in 71 (17%) cases.

In 33 (46%) of the cases, MCS was implanted before PCI (Upfront group), whereas in 38 (54%) of the patients, it was started at any point after the start of the procedure (Procedural group). In the Upfront group, the mean age was 67 ± 10 years, while in the Procedural group it was 62 ± 11 years (*p* = 0.05). Both groups were mainly represented by male patients (76% vs. 82%, respectively; *p* = 0.57). Baseline characteristics and cardiovascular risk factors (arterial hypertension, hyperlipoproteinemia, body mass index, diabetes mellitus), were found to be comparable between the two groups. Also, no significant differences were observed with regard to the history of the coronary artery interventions performed in patients with established coronary artery disease, namely a history of PCI (15% vs. 16%, respectively; *p* = 0.99) or coronary artery bypass grafting (6% vs. 5%, respectively; *p* = 0.99). The rate of out-of-hospital cardiac arrests was also similar in the Upfront and Procedural groups (25% vs. 28%, respectively; *p* = 0.99).

The majority of AMI patients presented with ST-elevation myocardial infarction (79% vs. 74%, respectively; *p* = 0.78) and most of them were already intubated (61% vs. 74%, respectively; *p* = 0.31). While all the patients had a running Noradrenaline infusion at presentation, there was no significant difference regarding dosages at the time of their admission (6.6 mL/h ± 6.2 mL/h vs. 8.5 ± 6.5 mL/h, respectively; *p* = 0.09) and after 24 h (7.2 mL/h ± 5.9 mL/h vs. 6.5 ± 3.4 mL/h, respectively; *p* = 0.88). Also, no significant difference was observed when considering the time from presentation to the delivery of MCS, the so-called “door to support” time (116 min ± 82 min vs. 102 min ± 79 min, respectively; *p* = 0.59) (Table 1).

**Figure 1:**
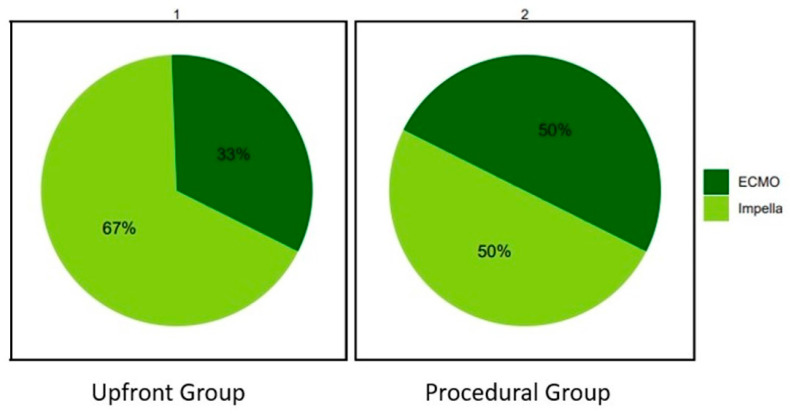
Distribution of mechanical circulatory supports’ usage in the Upfront and Procedural groups. ECMO: extracorporeal membrane oxygenation.

In the Upfront group, the Impella device was more frequently used compared to the ECMO (67% vs. 33%). In the Procedural group their proportions were similar (50% vs. 50%) (Figure 1).

The vast majority of patients had multivessel coronary artery disease (MVD), in both groups (82% vs. 84%, respectively; *p* = 0.99). However, an MVD-PCI was performed only in 45% and 42% of cases, respectively (*p* = 0.81). Accordingly, complete revascularization was achieved in 52% and 34% of cases, respectively (*p* = 0.16).

Complex coronary artery interventions, defined by extensive calcification, bifurcation lesions, large volumes of contrast use, or prolonged procedural time, were performed in the majority of cases in both groups. However, these were more frequent in the Upfront group (94% vs. 71%, respectively; *p* = 0.02). Lesions considered heavily calcified, requiring special lesion preparation techniques, were comparable (9% vs. 8%, respectively; *p* = 0.99), while more bifurcation PCIs were performed in the Upfront group (48% vs. 26%, respectively; *p* = 0.08). There were no significant differences regarding the median amount of contrast used (246 ± 98 mL vs. 252 ± 121 mL, respectively; *p* = 0.82) or the median duration of the procedure (142 ± 62 min vs. 134 ± 60 min, respectively; *p* = 0.59). The total length of the implanted stents was higher in the Upfront than in the Procedural group (66 ± 44 mm vs. 47 ± 31 mm, respectively; *p* = 0.05). There was only one case of vascular bleeding complication reported in the Procedural group and one case of ischemic complication in the Upfront group, both without statistical significance. The procedural characteristics are presented in Table 2. The total number of days spent in the coronary care unit, as well in the hospital, before death or discharge, were similar between groups. The outcomes are presented in Table 3.

**Figure 2:**
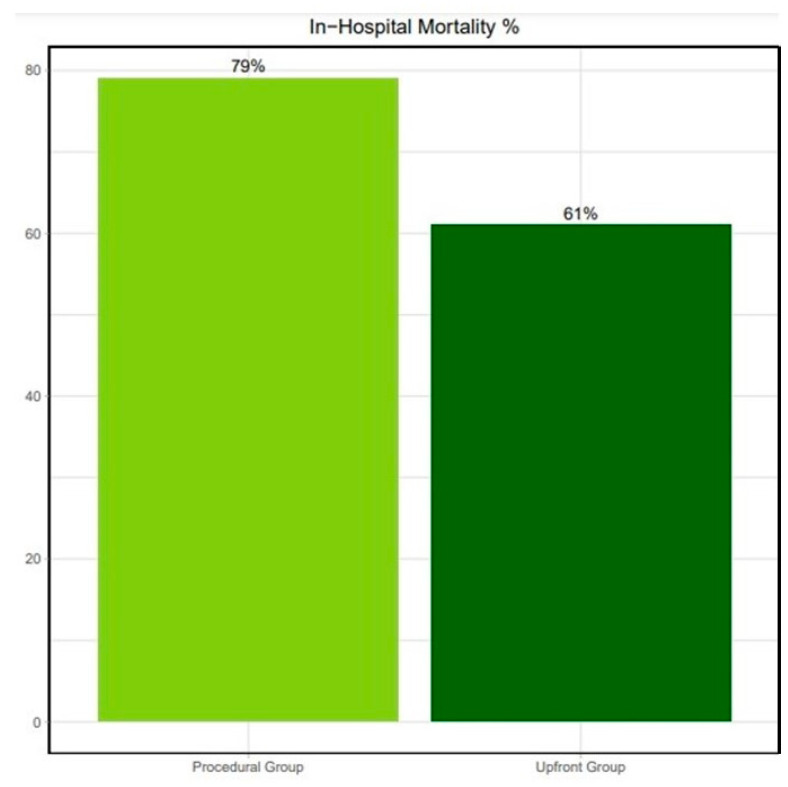
In-hospital mortality for the Upfront and Procedural groups.

**Figure 3:**
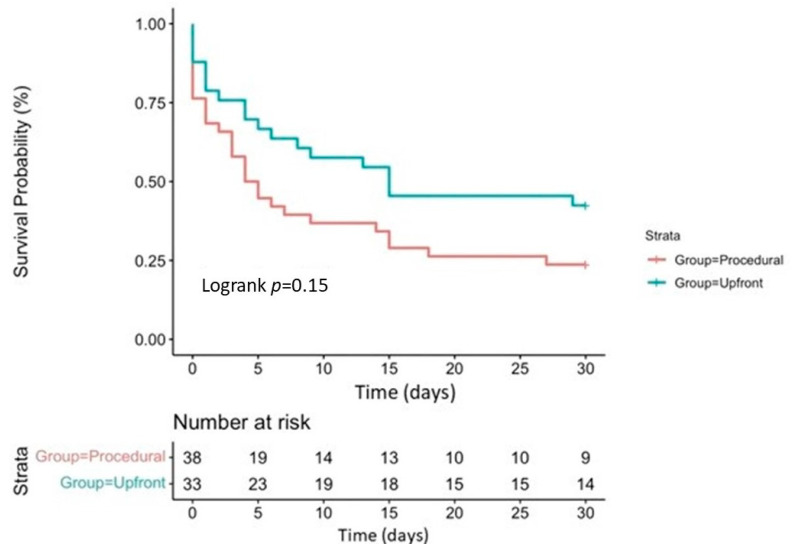
Kaplan–Meier survival curve for 30-day survival in the Upfront and Procedural groups.

When comparing patients with Upfront versus Procedural MCS, periprocedural CPR was significantly more frequent in the latter group (45% vs. 79%, *p* < 0.05). Still, in-hospital mortality remained similar in both groups (61% vs. 79%, respectively; odds ratio 1.55 [0.93 to 2.46]; *p* = 0.12); Figure 2. Their 30-day survival was also comparable (39% vs. 21%, respectively; *p* = 0.12); all the patients discharged from our center were alive at their 1-month follow-up; Figure 3.

## 4. Discussion

The present data, which include similar proportions of patients who received a device either before or after the start of their PCI, suggest that timing of MCS implantation has no impact on the in-hospital survival of patients with myocardial infarction-related CS.

Given these findings, coupled with the absence of definitive evidence or clear guidelines for MCS in AMI-related CS [13], several questions naturally arise that could influence decision making in everyday practice. Firstly, the impact of MCS intervention on the acute phase of CS and its subsequent effects on prognosis and mortality remain uncertain. Secondly, the optimal timing for MCS deployment is still undetermined, with a lack of clear guidance in the existing literature. Lastly, despite the fact that the IABP was largely abandoned after 2012, choices for the best available device [14,15] still remain unclear. In light of these uncertainties, MCS currently holds a Class IIa recommendation in European Guidelines, while its use has decreased in the last decade, indicating a decline in routine MCS use and the need for careful patient selection [13,16].

Multiple RCTs have addressed the question of a potential benefit to the use of MCS devices over medical therapy alone in AMI patients [5,6,17], especially in a CS setting. This scenario involves complex pathophysiologic conditions beyond a low cardiac output, which can include consequential inflammatory responses or irreversible advanced organ failure that could prove to not be reversible with mechanical circulatory support alone [18].

In the large, randomized, prospective, multicenter IABP-SHOCK trial, the use of IABPs did not manage to significantly reduce the 30-day mortality rate in patients presenting with CS-complicating AMI, for which an early revascularization procedure was planned [5]. The study showed a shift from IABP to Impella CP. Also, patients receiving the IABP were excluded from our registry. The landmark ECLS-SHOCK trial, which addressed the impact of extracorporeal life support on mortality in patients presenting with MI complicated by CS, failed to demonstrate a benefit of MCS compared to medical therapy alone for the composite endpoint of death from any cause, resuscitated cardiac arrest, and the need for the delivery of a supplementary MCS after 30 days [6]. Also, no significant differences were detected when considering safety endpoints, with the trial emphasizing the increased incidence of device-related complications, such as limb ischemia or severe bleeding, which may counter the potential hemodynamic benefits of MCS in this category of patients. Notably, over one third of the patients from the conservative group crossed over and received an MCS device. Furthermore, when the safety data are interpreted, it should be noted that the occurrence of safety issues and complications, such as the ones mentioned above, increase proportionally with the duration of ECMO treatment. In our center’s experience, there has been only one reported case of severe bleeding complication at the access site. This occurred in a patient with severe disseminated intravascular coagulation, complicating the initial scenario of concomitant sepsis and acute myocardial infarction-related cardiogenic shock. In such a complex setting, categorizing this complication as being solely related to the MCS (mechanical circulatory support) device implantation procedure would be challenging. Additionally, an acute limb ischemia was described in a case of critical peripheral arterial disease. Both situations underline the importance of the decision-making process and attentive patient selection, while balancing the expected outcomes and procedural risks of the procedure.

On the other hand, Kapur et al. highlight the potential of MCS not only to support hemodynamics but also to directly modulate the cellular pathways which are involved in myocardial injury and repair. In the context of MCS use, their study shows an increased phosphorylation of the reperfusion injury salvage kinase pathway, indicating a potential mechanism through which MCS may confer myocardial protection [19]. Among the benefits of MCS for myocardial salvage, the reduction in myocardial oxygen consumption, coupled with nearly normal lactate extraction ratios, correlates with improved subendocardial blood flow during reperfusion and infarct size reduction [20].

These contradictory findings are thus challenging the assumption that an early delivery of MCS devices in AMI-CS patients would provide immediate hemodynamic support and improve their outcomes, a hypothesis which aligns with the findings of our study. This could suggest the need for tailoring the indications for MCS based on the severity of CS or on individual patient profiles, and not only on a predefined timing strategy. Moreover, higher survival rates were associated with centers that had greater volumes of Impella use, suggesting that institutional experience plays a critical role in optimizing MCS outcomes [21].

The timing of MCS implementation, either before or after the PCI procedure, has demonstrated no impact on in-hospital survival [13], which is also the main finding of the present study. This evidence generates several questions concerning the optimal timing for deployment of such devices to impact outcomes. The same uncertainty was underlined by the IMPRESS in the STEMI trial, where the timing of MCS implantation had no impact on in-hospital mortality or survival, with further no reduction in the 30-day mortality of percutaneous MCS when it was compared to IABP [18]. Mortality rates were also very high in this study, surpassing those in the IABP-SHOCK trial. The authors suggest a lack of mortality improvement with MCS due to an unselective inclusion of patients that might potentially have had post-anoxic neurological damage at randomization. This clinical scenario could also apply to the real-world unrandomized population included in our study.

Smaller studies suggest a potential benefit of and an association between the early utilization of MCS and improved early hemodynamics, survival rates, and prognosis in patients presenting with AMI-CS [19,20]. The benefits of early MCS for myocardial salvage consist not only of unloading the left ventricle and increasing diastolic blood pressure, but also in a decrease in metabolic demand, especially in the initial hours of reperfusion or, ideally, during ischemia, with a subsequent reduction in the myocardial infarct’s size [20]. Moreover, a reduction in mortality was reported when MCS was initiated within 90 min after the onset of the cardiogenic shock, before the administration of inotropic support or performing a PCI, with a significant increase in survival [22,23]. These findings emphasize the critical window for intervention that could maximize the benefits of MCS in this category of patients. By doing so, they support the efficacy and feasibility of early MCS across diverse healthcare settings by providing important amelioration in perfusion and hemodynamics [23]. Notably, as the impact of MCS on hemodynamics can vary, the pulmonary artery catheter (PAC), as a tool for hemodynamic monitoring, could guide the management and also the escalation of MCS, which may involve switching to a higher-flow or even adding a second device [24]. Although its use in CS patients is still controversial, recent retrospective studies suggest that PAC could improve the outcomes in CS patients who have already received an Impella pump [21]. In our center, the PAC is not routinely used in the catheterization laboratory, nor on the intensive care unit for providing hemodynamic data. Instead, other invasive and noninvasive modalities of hemodynamic monitoring, such as peripheral arterial catheterization, serial echocardiography, or biomarker assessment, are utilized for the continuous monitoring of blood pressure, cardiac output, and the guiding of treatments in CS scenarios.

A further retrospective study conducted on 64 AMI-CS patients, randomized to receive IABP or Impella, showed that patients receiving Impella pumps before their PCI procedure experienced a reduction in infarct size as well as an improved myocardial recovery at their 6-month follow-up [25]. The same study highlighted the importance of an early MCS strategy in reducing reperfusion injury and left ventricular wall stress post AMI, while reducing the need for high-dose inotropes. These advantages were particularly evident when accompanied by a lower rate of MCS-related complications, highlighting not only the immediate hemodynamic benefits of MCS but also its long-term impact on myocardial recovery and function.

For patients presenting with AMI-related CS, early diagnosis of the condition and a short time to coronary reperfusion, the so-called “door to support” time, analogous to the “door to balloon” time in STEMI patients or to the ‘time-is-muscle’ paradigm [26], are key for improving survival and have become routine in management workups [27]. This approach emphasizes the importance of minimizing the time from hospital admission to MCS deployment to improve outcomes in CS management. Since a reduction in all-cause mortality was observed due to the implementation of MCS devices pre-PCI, or even before the initiation of vascular resuscitation, actual studies support the concept of a “door to support” time in ACS-CS patients [28]. Nevertheless, in our center’s experience, there is no significant difference in the “door to support” time between the patients who received MCS before or after the start of their PCI procedure.

The evidence presented indicates a shift towards recognizing the potential benefits of early MCS initiation in the management of AMI-CS, which underlines the need for clear guidelines and standardized protocols to optimize the timing of MCS deployment in clinical practice. Additionally, by exploring the mechanisms by which MCS devices impact myocardial recovery and long-term function, valuable insights into optimizing device delivery timing could be provided.

## 5. Limitations

An important limitation to our study was its relatively small number of patients. Given its non-randomized nature, device selection as well as the timing of MCS delivery were at the discretion of the operators, resulting in certain treatment biases. Also, our dataset has limited variables in order to formulate a clear distinction between SCAI stages C and D and thus accurately assess baseline to maximal SCAI classes.

## 6. Conclusions

Our real-life dataset did not show statistical benefit in terms of in-hospital mortality when MCS was introduced prior coronary intervention.

## Figures and Tables

**Table 1:** Characteristics at presentation. ACS: acute coronary syndrome; BMI: body mass index; CABG: coronary artery bypass graft; CPR: cardiopulmonary resuscitation; NSTEMI: non-ST-elevation myocardial infarction; OHCA: out-of-hospital cardiac arrest; PCI: percutaneous coronary intervention; PaO_2_: Partial pressure of oxygen; pH: potential of hydrogen; STEMI: ST-elevation myocardial infarction.

	Upfront Group	*n* = 33	Procedural Group	*n* = 38	*p*
	*n*/Mean	% or SD	*n*/Mean	% or SD	
Age	67	±10	62	±11	0.05
OHCA	8	25	10	28	0.99
Any CPR	15	45	30	79	<0.05
Female gender	8	24	7	18	0.57
BMI	28	5	28	4	0.75
Hypertension	19	58	16	42	0.24
Dyslipidemia	11	33	8	21	0.29
Diabetes mellitus	6	18	11	29	0.40
History of PCI	5	15	6	16	0.99
History of CABG	2	6	2	5	0.99
ACS STEMI	26	79	28	74	0.78
ACS NSTEMI	7	21	10	26	0.78
Intubated before admission	20	61	28	74	0.31
Thrombolysis before admission	0	0	2	5.2	0.49
Noradrenalin (mL/h when 12.5 mg/50 mL)	6.6	6.2	8.5	6.5	0.09
Systolic blood pressure (mmHg)	95.7	20	95.9	23.7	0.85
Heart rate/minute	74.4	18.5	78.1	23.2	0.71
PaO_2_ (mmHg)	106.8	80.6	92.7	64.1	0.22
pH	7.20	0.21	7.24	0.15	0.36
Lactate level (mmol/L)	5.7	4.9	6.4	4.6	0.24
“Door to support” time (Minutes)	116	82	102	79	0.56

**Table 2:** Procedural characteristics. ECMO: extracorporeal membrane oxygenation; MV: multivessel; PCI: percutaneous coronary intervention.

	Upfront Group	*n* = 33	Procedural Group	*n* = 38	*p*
	*n*/Mean	% or SD	*n*/Mean	% or SD	
Multivessel Disease	27	82	32	84	0.99
MV Disease PCI	15	45	16	42	0.81
Full Revascularization	17	52	13	34	0.16
Complex PCI Procedure	31	94	27	71	0.02
Bifurcation Lesion PCI	16	48	10	26	0.08
Relevant Coronary Calcification (with need for special lesion preparation)	3	9	3	8	0.99
Contrast Used (mL)	246	98	252	121	0.82
Procedure Duration (Minutes)	142	62	134	60	0.59
Vascular Complications or Bleeding	0	0	1	3	0.99
Ischemic Complications	1	3	0	0	0.46
Use of IMPELLA	22	67	19	50	
Use of ECMO	11	33	19	50	0.23
In-Hospital Mortality	20	61	30	79	0.12

**Table 3:** Outcomes. CCU: coronary care unit; MCS: mechanical circulatory support.

	Upfront Group	*n* = 33	Procedural Group	*n* = 38	*p*
	*n*/Mean	% or SD	*n*/Mean	% or SD	
CCU Stay (Days)	9.8	10.2	8.6	9.8	0.5
Total Hospital Stay (Days)	14.5	14	13.6	20.4	0.26
Days on MCS	3.5	3.9	4.1	4.1	0.21
30-day Survival	13	40	8	21	0.12

## Data Availability

The data presented in this study are available on request from the corresponding author. The data are not publicly available due to privacy restrictions.
